# Molecular Phylogenetic Analysis of *16S rRNA* Sequences Identified Two Lineages of *Helicobacter pylori* Strains Detected from Different Regions in Sudan Suggestive of Differential Evolution

**DOI:** 10.1155/2020/8825718

**Published:** 2020-10-27

**Authors:** Abeer Babiker Idris, Hadeel Gassim Hassan, Maryam Atif Salaheldin Ali, Sulafa Mohamed Eltaher, Leena Babiker Idris, Hisham N. Altayb, Amin Mohamed Abass, Mustafa Mohammed Ahmed Ibrahim, El-Amin Mohamed Ibrahim, Mohamed A. Hassan

**Affiliations:** ^1^Department of Medical Microbiology, Faculty of Medical Laboratory Sciences, University of Khartoum, Khartoum, Sudan; ^2^The Academy of Health Sciences, The Republic of Sudan Federal Ministry of Health, Khartoum, Sudan; ^3^Faculty of Medicine, The International University of Africa, Khartoum, Sudan; ^4^Department of Biochemistry, College of Sciences, King Abdulaziz University, Jeddah, Saudi Arabia; ^5^Department of Internal Medicine, University of Bahri, Khartoum, Sudan; ^6^Consultant Physician and Gastroenterology, Khartoum, Sudan; ^7^Department of Bioinformatics, Africa City of Technology, Khartoum, Sudan; ^8^Department of Bioinformatics, DETAGEN Genetic Diagnostics Center, Kayseri, Turkey; ^9^Department of Translation Bioinformatics, Detavax Biotech, Kayseri, Turkey

## Abstract

**Background:**

*Helicobacter pylori* (*H. pylori*) is ubiquitous among humans and one of the best-studied examples of an intimate association between bacteria and humans. Phylogeny and Phylogeography of *H. pylori* strains are known to mirror human migration patterns and reflect significant demographic events in human prehistory. In this study, we analyzed the molecular evolution of *H. pylori* strains detected from different tribes and regions of Sudan using *16S rRNA* gene and the phylogenetic approach. *Materials and methods*. A total of 75 gastric biopsies were taken from patients who had been referred for endoscopy from different regions of Sudan. The DNA extraction was performed by using the guanidine chloride method. Two sets of primers (universal and specific for *H. pylori*) were used to amplify the *16S ribosomal* gene. Sanger sequencing was applied, and the resulted sequences were matched with the sequences of the National Center for Biotechnology Information (NCBI) nucleotide database. The evolutionary aspects were analyzed using MEGA7 software.

**Results:**

Molecular detection of *H. pylori* has shown that 28 (37.33%) of the patients were positive for *H. pylori* and no significant differences were found in sociodemographic characteristics, endoscopy series, and *H. pylori* infection. Nucleotide variations were observed at five nucleotide positions (positions 219, 305, 578, 741, and 763–764), and one insertion mutation (750_InsC_751) was present in sixty-seven percent (7/12) of our strains. These six mutations were detected in regions of the *16S rRNA* not closely associated with either tetracycline or *tRNA* binding sites; 66.67% of them were located in the central domain of *16S rRNA*. The phylogenetic analysis of *16S rRNA* sequences identified two lineages of *H. pylori* strains detected from different regions in Sudan. The presence of Sudanese *H. pylori* strains resembling Hungarian *H. pylori* strains could reflect the migration of Hungarian people to Sudan or vice versa.

**Conclusion:**

This finding emphasizes the significance of studying the phylogeny of *H. pylori* strains as a discriminatory tool to mirror human migration patterns. In addition, the *16S rRNA* gene amplification method was found useful for bacterial identification and phylogeny.

## 1. Introduction

*Helicobacter pylori (H. pylori)* is ubiquitous among humans [1] and one of the best-studied examples of an intimate association between bacteria and humans [2]. It has infected human around 100,000 years ago (range: 88,000–116,000) [1, 3] and has coevolved with humans ancestors for approximately 58000 ± 3500 years, during their first migrations from east Africa [1, 4, 5]. Also, it largely escaped notice until it was cultured by Marshall and Warren [6, 7]. In fact, *H. pylori* possesses several properties that help this bacterium to persist for several decades, transmit from generation to generation, and make an intimate association with its human host [2, 8]. *H. pylori* infection is predominantly transmitted vertically from the parent to child and between individuals in close contact such as in a family [2, 9]. This close transmission pattern has resulted in a clear phylogeographic differentiation within these bacteria because of the local dispersion of single nucleotide polymorphisms by high rates of homologous recombination [10, 11]. However, under poverty and inappropriate conditions, especially in the developing countries, *H. pylori* can be transmitted horizontally which makes infection with multiple strains probably more common than in the developed world [12, 13].

In Sudan, the population is culturally, linguisticall,y and ethnically diverse with more than 597 tribes who speak more than 400 dialects and languages [14, 15]. The majority of the Sudanese population is rural with an urban population of just 33.2%; most of them are in Khartoum [15, 16]. Sudan has severely suffered war, famine, and flood in recent decades and has a large population of internally displaced persons (IDPs) [17, 18]. Although Sudan is rich in terms of natural and human resources, the effects of the civil conflict on health, nutrition, population, and economic and social development have undoubtedly been significant. These several diverse sociodemographic factors lead to a continued increase in the prevalence rate of *H. pylori* infection ranging from 48% to 65.8% which represents a major public health challenge [19, 20].

However, studying the genetics of the *H. pylori* population has been of great interest due to its clinical and phylogeographic significance [4]. A number of studies have discovered the importance of evolutionary history to the clinical outcome of *H. pylori* infection and indicated that the scramble of the relationship between bacterial and human ancestries at the individual level due to a consequence of migrations, invasions, or racial admixture may moderate adverse outcomes for the host and disrupting the selection for a reduced virulence; and this may give some explanation to the continental enigmas of *H. pylori* [13, 21–24]. Also, phylogeny and phylogeography of *H. pylori* strains are known to mirror human migration patterns [25–28] and reflect significant demographic events in human prehistory [26, 29]. Therefore, the phylogeographic pattern of *H. pylori* is a discriminatory tool to investigate human evolution and migration in addition to the traditional human genetic tools, e.g., mitochondrial DNA (mtDNA) and languages [1].

*16S ribosomal RNA (16S rRNA)* gene amplification and sequencing have been extensively used for bacterial phylogeny and taxonomy and, eventually, the establishment of large public-domain databases [30–33]. Several properties of the *16S rRNA* gene make it the “ultimate molecular chronometer” [34], the most common housekeeping genetic marker, and hence, a useful target for clinical identification and phylogeny [35, 36]. These properties include the following: First, it is present in all bacteria, often existing as a multigene family or operons; thus, it is a universal target for bacterial identification [33, 36, 37]. Second, the function of *16S rRNA* has not changed over a long period, so random sequence changes are more likely to reflect the microbial evolutionary change (phylogeny) than selected changes which may alter the molecule's function [34]. Finally, the *16S rRNA* gene is large enough, approximately 1,500 bp, for informatics purposes [35, 36, 38]. Most importantly, the *16S rRNA* gene consists of approximately 50 functional domains and any introduction of selected changes in one domain does not greatly affect sequences in other domains, i.e., less impact selected changes have on phylogenetic relationships [36].

Here, in this study, two sets of primers (universal and specific for *H. pylori*) were used to amplify the *16S ribosomal* gene directly from gastric endoscopic biopsy samples collected from dyspeptic patients who had been referred for endoscopy. Sanger sequencing was performed, and by matching these sequences with those available in the National Center for Biotechnology Information (NCBI) nucleotide database, we analyzed the evolutionary aspects of Sudanese *H. pylori* strains using a phylogenetic approach. The novelty of our study resides in being the first study to characterize *H. pylori* Sudanese strains detected from different regions and tribes of Sudan using the *16S rRNA* gene.

## 2. Materials and Methods

### 2.1. Study Design and Study Settings

A cross-sectional hospital-based study was conducted in public and private hospitals in Khartoum state from June 2018 to September 2019. These hospitals receive patients from all over the regions of Sudan. Currently, there are 16 states in Sudan which are divided between four geographic regions: Eastern, Western, Northern, and Central Sudan which includes the largest metropolitan area, Khartoum (that includes Khartoum, Khartoum North, and Omdurman) [39]. The capital Khartoum is quickly growing and populated with 6 to 7 million, which includes approximately 2 million IDPs from the southern war zone and the drought-affected areas in the west and east [15, 18].

The hospitals include Ibin Sina specialized hospital, Soba teaching hospital, Modern Medical Center, and Al Faisal Specialized Hospital. All laboratory processes were performed in the molecular biological laboratory at the Faculty of Medical Laboratory Sciences at the University of Khartoum.

### 2.2. Study Population

The study population composed of 75 patients who had been referred for endoscopy, and most of them were because of dyspepsia. The structured questionnaire, modified from [20], was provided for participants to obtain information about their sociodemographic and clinical characteristics. Patients were selected from those who were not taking antibiotics or nonsteroidal anti-inflammatory drugs (NSAIDs). The patients gave written informed consent before they enrolled in the study. The diagnosis of gastroduodenal diseases was based on the investigation of an experienced gastroenterologist during the upper gastrointestinal (GI) endoscopy procedure, while gastric cancer was diagnosed based on, in addition to gastroscopy, histology.

### 2.3. Sample Collection

For DNA extraction purposes, gastric biopsies were collected in 1.5 *µ*l Eppendorf tubes with 400 *µ*l phosphate buffer saline (PBS), while for histological examination, the biopsies were transported in 10% formalin. Then, the biopsies were labeled and transported immediately to the laboratory for further processes.

### 2.4. DNA Extraction

The DNA extraction was performed by using the manual guanidine chloride method [40, 41]. Briefly, biopsies were washed with 400 *µ*l PBS and centrifuged for 5 min at 1000 rpm after each wash. Then, the samples were subjected to digestion by adding 400 *µ*l of WBCs lysis buffer, 200 *µ*l of 6 M guanidine chloride, 50 *µ*l of 7.5 M ammonium acetate, and 5 *µ*l of 20 mg/*µ*l proteinase K and incubated at 37°C overnight. On the following day, samples were cooled down to room temperature; then, 400 *µ*l of cooled prechilled chloroform was added and centrifuged at 1000 rpm for 5 min. Then, three layers were separated, and the supernatant was collected to a new labeled Eppendorf. One ml of cooled prechilled absolute ethanol was added and mixed gently back and forth quickly. Samples were put in −20°C freezer overnight. After that, samples were subjected to quick vortex for one minute and, then, centrifuged for 5 min at 1000 rpm; and the supernatants were discarded. Then, washing with 70% ethanol was performed, and the supernatant was drained with much care to avoid losing the DNA pellet at the bottom of the Eppendorf. The Eppendorf was inverted upside down of a tissue paper leaving the pellet to dry from alcohol for, at least, 2 hours. Finally, the DNA pellet was resuspended in 35 *µ*l of deionized water and was put into −20°C until use. All the used chemical reagents were obtained from iNtRON Biotechnology Inc., Korea.

### 2.5. *16S rRNA* Gene Amplification

Extracted DNA was used to amplify the universal *16S rRNA* gene using the following primers: (primers: F:5′-AGAGTTTGATCCTGGCTCAG-3′) (R:5′-CTACGGCTACCTTGTTACGA-3′). PCR amplification was carried out with the Maxime PCR PreMix Kit (i-Taq) (iNtRON Biotechnology, Seongnam, Korea) and a PCR thermocycler (SensoQuest, Germany). The PCR reaction mixtures contained 2.5 U of i-Taq TM DNA polymerase (5 U/*µ*l), 2.5 mM of each deoxynucleoside triphosphates (dNTPs), 1X of PCR reaction buffer (10X), 1X of gel loading buffer, and 1 *µ*l of DNA template. The temperature cycle for the PCR was carried out using a method described previously [42].

To detect the DNA, 3 *µ*l of each PCR products was loaded onto 2% agarose gels stained with 3 *µ*l ethidium bromide (10 mg/ml) and subjected to electrophoresis in 1x Tris EDTA Buffer (TEB buffer) (89 mM of Tris base, 89 mM Boric acid, and 2 mM EDTA dissolved in 1 Litter H_2_O) for 30 min at 120 V and 50 mA. The gel was visualized under UV light illumination. A 100 bp DNA ladder (iNtRON Biotechnology, Seongnam, Korea) was used in each gel as a molecular size standard. The amplified product for the *rrs* gene is 1500 bp.

The DNA amplification for the specific *16S rRNA* genes was performed to confirm the infection of *H. pylori* (primers: F:5′-GCGCAATCAGCGTCAGGTAATG-3′) (R:5′-GCTAAGAGAGCAGCCTATGTCC-3′) [43]. The PCR reaction used was an initial step of 3 min at 94°C, followed by denaturation for 30 sec at 94°C, annealing for 30 sec at 53°C, and primer extension for 45 sec at 72°C. After the 40th cycle, the final extension step was prolonged for 5 min to complete the synthesis of strands. The amplified product for the specific *rrs* gene is 522 bp.

### 2.6. Sequencing of *H. pylori 16S rRNA* Gene

The amplified *16S rRNA* gene (for the universal and specific) was purified and sequenced, using the Sanger dideoxy sequencing method, commercially by Macrogen Inc, Korea.

### 2.7. Bioinformatics Analysis

#### 2.7.1. Sequence Analysis

The nucleotide sequence was visualized and analyzed by using the Finch TV program version 1.4.0 [44]. The nucleotide Basic Local Alignment Search Tool (BLASTn; https://blast.ncbi.nlm.nih.gov/) was used for searching about the similarity with other sequences deposited in GenBank [45]. The *16S rRNA* genes sequences were submitted in the GenBank nucleotide database under the following accession numbers: from MN845181 to MN845190 and from MN845952 to MN845954.

#### 2.7.2. Molecular Phylogenetic Analysis

Highly similar sequences were retrieved from the NCBI GenBank and subjected to multiple sequence alignment (MSA) using Clustal W2 [46]-BioEdit software [47]. Gblocks was used to eliminate poorly aligned positions and divergent regions of aligned sequences so the alignment becomes more suitable for phylogenetic analysis [48, 49]. The Neighbor-Joining phylogenetic tree [50] of our *16S rRNA* sequences with those obtained from the database was constructed using the Jukes–Cantor (JC) model [51] from the substitution (ML) model. [52] The tree was replicated 1000 replicates in which the association with taxa clustered together in the bootstrap test [53]. Molecular Evolutionary Genetics Analysis Version 7.0 (MEGA7) was used to conduct evolutionary analyses [54].

### 2.8. Statistical Analysis

Data were analyzed using the GraphPad Prism 5. Regarding the prevalence of *H. pylori* infection, differences in frequency distribution by age were examined by the Mann–Whitney test. While bivariate analysis with a categorical variable was assessed by the *χ*^*2*^ test or Fisher's test. The statistical significance level was determined at *P* < 0.05.

## 3. Results

### 3.1. Characteristics of the Study Population

**Figure 1:**
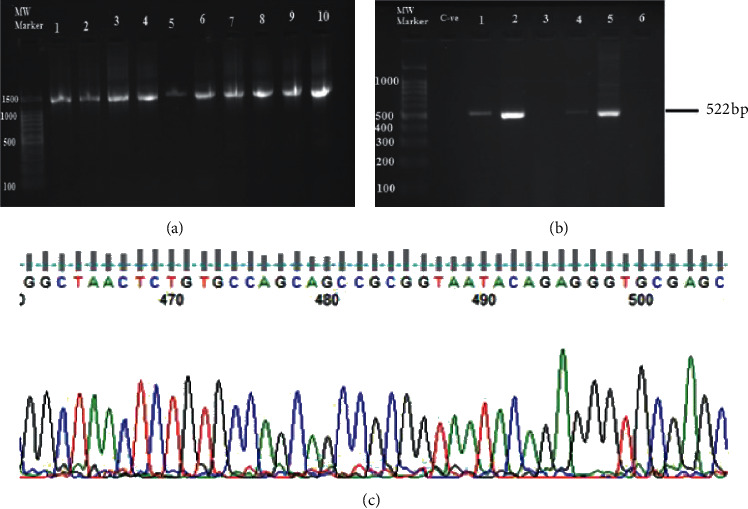
(a) and (b) PCR amplification results of universal and specific *H. pylori* 16S rRNA gene, respectively, examined on 2% agarose gel electrophoresis. (c) Sequencing result of *Acinetobacter radioresistens* chromatogram using Finch TV software. The nucleotide sequence was deposited in the GenBank database under the accession number MN845952.

A total of seventy-five patients were included. Forty-one patients (54.67%) were males, and thirty-four (45.33%) were females. Forty-two patients (56%) were urban, and thirty-three (44%) were rural residents. The patients' age ranged from 15 to 85 years, with a mean age of 45.11 ± 17.45 years (Table 1). Most of the participants came from Northern and Central Sudan. Also, their ethnicities were distributed as follows: Shagia (10, 13.33%), Jalyeen (9, 12%), Mahas (8, 10.67%), Rezaigat (4, 5.33%), Zaghawa (4, 5.33%), Kawahla (4, 5.33%), Masalamyia (3, 4%), and others (33, 44%); see Table 1. Regarding clinical symptoms of gastrointestinal disturbances of the participants, abdominal pain was the major symptom (22, 29.33%), followed by nausea (16, 21.33%), and the incidence of *H. pylori* is more common in patients with gastritis (28.57%) (Table 2). Molecular detection of *H. pylori* has shown that 28 (37.33%) of patients were positive for *H. pylori* (Figure 1). Patients from Western Sudan were more prone to *H. pylori* infection 50% (6/12). Bivariate analysis has found that no significant differences were exhibited across sociodemographic, endoscopy series, and *H. pylori* infection, as illustrated in Tables 1 and 2.

### 3.2. Analysis of *16S rRNA* Sequences

**Figure 2:**
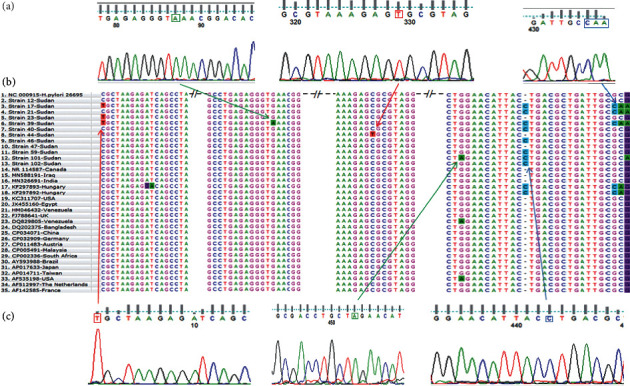
(a) and (c) Sequencing results of chromatograms using Finch TV software show nucleotide variations in the 16S rRNA gene of *H. pylori* illustrated by squares. (b) Multiple Sequence Alignment (MSA) of 16S rRNA sequences of 12 Sudanese *H. pylori* strains compared with the rrnA gene of *H. pylori* strain 26695 (NC_000915) and other selected strains obtained from GenBank databases using Clustal W2.

Twelve sequences of *16S rRNA of H. pylori* from Sudanese patients were analyzed for mutations and their conservative nature; and findings revealed diversity with few differences, see Table 3 and Figure 2. The description of Sudanese *H. pylori* strains is shown in Table 3. The amplicon of universal *16S rRNA* for patients 22 resulted in *Acinetobacter radioresistens* (Figure 1). The patient 22 was male with age in the range of [16–36] years, he was Mahasi from Northern Sudan, and he was diagnosed with antral gastritis.

Regarding mutations, nucleotide variations were found at five nucleotide positions (positions 219, 305, 578, 741, and 763-764), and one insertion mutation (750_InsC_751) was present in sixty-seven percent (7/12) of our strains; the numbering was according to the *E.coli* sequence [55]. Three strains (17, 21, and 39) revealed a novel transition C⟶T at position 219. A triple base pair substitution (GCC763-764CAA) was detected in strain 17, 23, and 39. However, a cancerous strain 101 had two G⟶A transitions at positions 741 and 765. Strain 44, which was detected in a patient with normal gastric finding and suspected celiac disease and lives in Khartoum, also had a novel C⟶T transversion at nucleotide position 578. Also, strain 39, which was found in a patient from the West of Sudan (Eastern Darfur) and was diagnosed with antral gastritis, revealed a novel G⟶A transition at positions 305. See Figure 2 for more illustration.

### 3.3. Phylogenetic Analysis

**Figure 3:**
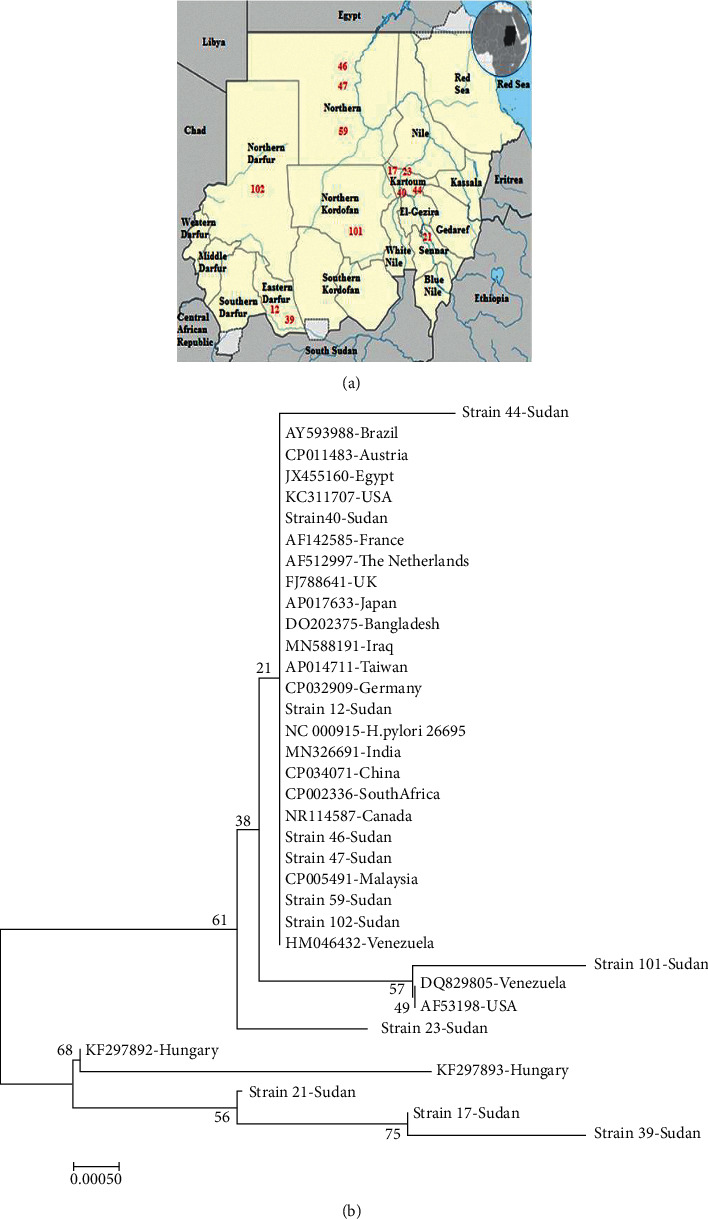
Distribution and evolutionary relationships of Sudanese *H. pylori* strains. (a) Map of the regional origin of strains in Sudan. (b) The Neighbor-Joining Phylogenetic tree. The percentage of replicate trees (1000 replicates) is shown next to the branches. The evolutionary distance was computed using the JC method and is in the units of the number of base substitution per site. Evolutionary analyses were conducted using MEGA7.

The phylogenetic tree diverged into two lineages (Figure 3). In the lineage one, all the Sudanese *H. pylori* strains clustered with strains from different countries. The *16S rRNA* sequence of strain 44, although clustered with other global strains in one clade, had a novel C⟶T transversion at nucleotide position 578. However, cancerous strain 101 shared a common ancestor with strains from the USA (AF535198) and Venezuela (DQ829805) and represented with them a separated clade with a bootstrap value of 57%. A novel single nucleotide variation (G⟶A) at position 305 of the *16S rRNA* of strain 23 made it outgroup as a kind of strain evolution with a bootstrap value of 61%.

In the second lineage, strains 17, 23, and 39 were closely related to strains from Hungary (KF297893 and KF297892). Strain 17 and Strain 39, from Khartoum and Eastern Darfur, respectively, were sisters with a bootstrap value of 75%, as presented in Figure 3.

## 4. Discussion

In this study, the phylogenetic analysis of *16S rRNA* sequences identified two lineages of *H. pylori* strains detected from different regions in Sudan which suggested differential evolution. This finding is in agreement with a number of studies that found an unusually high degree of genetic diversity in genomic sequence analyses of *H. pylori* strains in connection with their housekeeping and virulence-associated genes [26, 56, 57]. Strain 17, Strain 21, and Strain 39 were derived from one lineage along with two strains from Hungary. They shared a double base pair substitution (GC763-764CA) and one insertion mutation (751_InsC_752). Both mutations were located in the central domain of the *16S rRNA* gene. The presence of Sudanese *H. pylori* strains resembling Hungarian *H. pylori* strains could reflect the migration of Hungarian people to Sudan or vice versa. In 1935, Almásy and his colleague von der Esch became the first Europeans to re-establish contact with the Magyarab tribe [58]. Magyarab name is a concatenation of “Magyar” (Hungarian) and “ab” which means in Nubian simply “tribe.” So the name of the tribe combined translates to “Tribe of the Magyars” [59]. However, Hungarian ancestries came to Sudan in the late 16th century as a part of the Ottoman Empire army. Some of the Hungarian troops were sent to Wadi Halfa, a place located in the Northern state of Sudan. A portion or the entirety of them remained there and intermarried with local Nubian women, generating Magyarab tribe who have lived there ever since [60, 61]. But, because of the construction of the Aswan Dam and the flooding of Lake Nasser, most of Magyarab's villages and the ancient city of Faras were submerged [62, 63]. Therefore, some of them were resettled to New Halfa in the Butana region, the area between Al-Khartoum and Blue Nile, and others spread in many directions throughout Sudan [63, 64]. This could explain, to some extent, the variation in the regional origin of the strains (Strain 17, Strain 21, and Strain 39) that resemble Hungarian strains (Table 3). On the other hand, a significant number of Sudanese, especially from Western State, has left to Europe seeking safety and better life [65, 66].

Laboratory diagnosis of *H. pylori* by the conventional cultural methodology and phenotypic identification tests is still less specific, time-consuming, incurs increased capital costs, needs highly skilled personnel, and also is difficult to diagnose reinfections [67]. However, molecular tests and PCR technology have been raised as a useful alternative means of bacterial identification, which may circumvent some of these difficulties [67, 68] but has limitations mainly contamination and inadequate sensitivity issues [69]. In this study, molecular detection of *H. pylori* has shown that 37.33% of patients were positive for *H. pylori* in comparison to other studies conducted in different regions of Sudan, 22.2%, 59%, 65.8%, 40.1%, 48%, and 21.8% by Abdalsadeg et al., respectively, using different detection methods [19, 20, 70–73]. The present study revealed that there was no statistically significant association between the incidence of *H. pylori* infection and age, gender, and residence (Table 1) which are in agreement with previous studies [20, 74–77]. However, some other studies have shown different results [78–82]. These contradictory findings may be attributable to the difference in sample size and studied population. [83] Moreover, no significant association (*P*=0.7718) between the rate of infection and history of smoking was found in this study, which is similar to previous studies [20, 80, 84]. Cigarette smoking stimulates the excretion of hydrochloric acid which is regarded as a natural protective barrier against infective microorganism such as *H. pylori* [85]. Although no significant differences were found in frequency distribution of *H. pylori* infection by tribes or regions of participants, patients from Western Sudan were more prone to *H. pylori* infection 6/12 (50%). This could be explained by the low socioeconomic status, sanitary conditions, and level of educational background caused by drought, war, and civil conflict in Western Sudan compared to other regions [18]. Also, the study showed no significant difference in endoscopy series and status of *H. pylori* infection; however, the incidence of *H. pylori* is more common in patients with gastritis (28.57%) which is not totally unexpected since the first presentation of the infection with *H. pylori* is a symptomatic gastritis [86].

Tetracycline is one of the 30S-targeting antibiotics that inhibit translation elongation by sterically interfering with the binding of aminoacyl-tRNA to the A-site of the ribosome [87]. Therefore, mutations located in two domains (III and IV) in *16S rRNA*: helix 34 and the loop next to helix 31 [88] can affect the conformation of the tetracycline-binding site, leading to high-level resistance [87]. In this study, six mutations were detected in two domains (I and II) which are in regions of the *16S rRNA* not closely associated with either tetracycline or tRNA binding sites. This finding is partially in agreement with a study conducted by Catharine et al., who found six nucleotide changes in two tetracycline-resistance strains. Two of these changes were located in domain I and domain II, G360 A and deletion of G771, respectively [89]. Thus, further studies for *in vitro* phenotypic resistance are encouraged to investigate the association between these mutations and phenotypic tetracycline-resistance and examine the reliability of these mutations as molecular markers for tetracycline-resistance. Furthermore, we observed that 66.67% of nucleotide variations were located in the central domain of *16S rRNA* (nucleotides 567–915) which is associated with five ribosomal proteins (S15, S6, and S18) folds into the platform of the small subunit [90–93]. The deleterious mutations (C18G, A55G, A161G, A373G, G521A, C614A, A622G, and deletion of one A in a triple-A cluster 607–609) in domains I and II which were suggested by Aymen et al., in purposes of revealing covered putative functional regions of the ribosome and aiding in the development of new antibiotics [94], were found conserved in our strains, as illustrated in Figure 2(b).

Interestingly, the insertion mutation (InsC) between two nucleotides (750 and 751), which was detected in sixty-seven percent (7/12) of our strains, is located at the lower portion of helix 22 (H22), which is one of the lower three-helix junctions (3HJ) of *16S rRNA* that bind with S15 [95, 96]. Also, the mutation in cancerous strain 101 (G ⟶ A transition at positions 741) was located in the upper portion of helix 22 (H22). The molecular dynamic (MD) simulations conducted by Wen et al. showed that *16S rRNA* and S15 bind across the major groove of H22 via electrostatic interactions, i.e., the negatively charged phosphate groups of G658, U740, G741, and G742 bind to the positively charged S15 residues Lys7, Arg34, and Arg37 [95]. However, studying the effect of these mutations on the functions of *16S rRNA* molecules in protein synthesis and antibiotic resistance is of great importance especially for essential regions such as the central domain. [97] For example, Prescott and Dahlberg in 1990 found that the presence of substitution from a C to G at position 726 induces the synthesis of heat shock protein and affects the expression levels of various proteins [98]. Also, mutations in the sequence 911–915 were conferred streptomycin-resistance by impaired the binding of this antibiotic [99–101].

We acknowledge that limitations of the study are the small sample size, and the phylogenetic tree was built based on the *16S rRNA* gene only which is unable to cover more complex evolutionary events and distinguish between closely related strains or species [4]. Therefore, further national studies with a large sample size are recommended; also, building a phylogeny by increasing the number of genes analyzed like multilocus sequence analysis (MLST).

### 4.1. Conclusions

In this study, the *16S rRNA* gene amplification method was found useful for bacterial identification and phylogeny. The positive *H. pylori* infection rate among participants was 37.33%, and no significant differences were found in sociodemographic, endoscopy series, and *H. pylori* infection. Regarding mutations, six nucleotide variations were detected in regions of the *16S rRNA* not closely associated with either tetracycline or tRNA binding sites; 66.67% of them were located in the central domain of *16S rRNA*. The phylogenetic analysis of *16S rRNA* sequences identified two lineages of *H. pylori* strains detected from different regions in Sudan. The presence of Sudanese *H. pylori* strains resembling Hungarian *H. pylori* strains could reflect the migration of Hungarian people to Sudan or vice versa. This finding emphasizes the significance of studying the phylogeny of *H. pylori* strains as a discriminatory tool to mirror human migration patterns.

## Figures and Tables

**Table 1:** Sociodemographic characteristic of patients.

Variables		Total *n* = 75	*H. pylori* (+ve) *n* = 28	*H. pylori* (−ve) *n* = 47	*P* value
Mean age (years) ± std. deviation (range)		45.11 ± 17.45 (15–85)	41.39 ± 17.57 (15–85)	47.32 ± 17.18 (21–80)	0.1170
Gender	Male	41 (54.67%)	15 (53.57%)	26 (55.32%)	1.0000
	Female	34 (45.33%)	13 (46.43%)	21 (44.68%)	
Residence	Urban	42 (56%)	15 (53.57%)	27 (57.45%)	0.8122
	Rural	33 (44%)	13 (46.43%)	20 (42.55%)	
Hospital	Public	51 (68%)	20 (71.43%)	31 (65.96%)	0.7986
	Private	24 (32%)	8 (28.57%)	16 (34.04%)	
History of previous infection	Yes	42 (56%)	15 (53.57%)	27 (57.45%)	0.8122
	No	33 (44%)	13 (46.43%)	20 (42.55%)	
The frequency of recurrence (if yes *n* = 42)	One time	32 (76.19%)	10 (66.67%)	22 (81.49%)	0.4508
	More than one time	10 (23.81%)	5 (33.3%)	5 (18.52%)	
Family history of infection	Yes	36 (48%)	15 (53.58%)	21 (44.68%)	0.4833
	No	39 (52%)	13 (46.43%)	26 (55.32%)	
Smoking	Yes	16 (21.33%)	5 (17.86%)	11 (23.4%)	0.7718
	No	59 (78.67%)	23 (82.14%)	36 (76.6%)	
Tribe	Shagia	10 (13.33%)	4 (14.29%)	6 (12.77%)	0.5733
	Jalyeen	9 (12%)	4 (14.29%)	5 (10.64%)	
	Mahas	8 (10.67%)	3 (10.71%)	5 (10.64%)	
	Rezaigat	4 (5.33%)	1 (3.57%)	3 (6.38%)	
	Zaghawa	4 (5.33%)	3 (10.71%)	1 (2.13%)	
	Kawahla	4 (5.33%)	2 (7.14%)	2 (4.26%)	
	Masalamyia	3 (4%)	2 (7.14%)	1 (2.13%)	
	Other	33 (44%)	9 (32.14%)	24 (51.06%)	
Region	Northern Sudan	15 (20%)	6 (21.43%)	9 (19.15%)	0.2697
	Central Sudan	31 (41.33)	12 (42.86%)	19 (40.43%)	
	Eastern Sudan	3 (4%)	2 (7.14%)	1 (2.13%)	
	Western Sudan	12 (16%)	6 (21.43%)	6 (12.77%)	
	Southern Sudan	14 (18.67%)	2 (7.14%)	12 (25.53%)	

**Table 2:** Distribution of participants according to endoscopy series and status of *H. pylori* infection.

Endoscopy results	No. (%)	*H. pylori* (+ve)	*H. pylori* (−ve)	*P* value
Normal gastric findings	9 (12%)	4 (14.29%)	5 (10.64%)	0.5259
Esophagitis	4 (5.33%)	3 (10.71%)	1 (2.13%)	
Esophageal varices	6 (8%)	1 (3.57%)	5 (10.64%)	
Gastritis	26 (34.67%)	8 (28.57%)	18 (38.3%)	
Duodenitis	6 (8%)	3 (10.71%)	3 (6.38%)	
Peptic ulcer	6 (8%)	3 (10.71%)	3 (6.38%)	
Cancer	18 (24%)	6 (21.43%)	12 (25.53%)	
Total	75 (100%)	28 (37.33%)	47 (62.67%)	

**Table 3:** Description of Sudanese *H. pylori* strains.

Strain	Age of the patient (in range)	Clinical origin	Ethnic origin	Province	Region	Accession no.
Strain 12	[16–36]	Antral gastritis	Rezaigat	Eastern Darfur	West of Sudan	MN845953
Strain 17	[16–36]	Distal esophagitis and antral gastritis	Halawyeen	Khartoum	Middle of Sudan	MN845187
Strain 21	[16–36]	Antral gastritis	Kawahla	Sinnar	Southeast of Sudan	MN845181
Strain 23	[16–36]	GRED	Manaseer	Kartoum	Middle of Sudan	MN845188
Strain 39	[37–61]	Antral gastritis	Masalamyia	Eastern Darfur	West of Sudan	MN845189
Strain 40	>60	Duodenitis	—	Khartoum	Middle of Sudan	MN845182
Strain 44	[16–36]	Normal gastric finding	Jalyeen	Khartoum	Middle of Sudan	MN845183
Strain 46	[37–61]	Duodenitis	Shagia	Northern State	North of Sudan	MN845184
Strain 47	[16–36]	GRED	Shagia	Northern State	North of Sudan	MN845954
Strain 59	[37–61]	Normal gastric finding	Shagia	Northern State	North of Sudan	MN845190
Strain 101	[37–61]	Gastric cancer	Zaghawa	North Kordofan	South of Sudan	MN845185
Strain 102	[37–61]	Gastric cancer	Zaghawa	Northern Darfur	East of Sudan	MN845186

## Data Availability

All data generated or analyzed during this study are included in this published article.
